# The interplay of EBV virus and cell metabolism in lung cancer

**DOI:** 10.1111/jcmm.70088

**Published:** 2024-11-27

**Authors:** Hongwei Wang

**Affiliations:** ^1^ Shanxi Province Cancer Hospital/Shanxi Hospital Affiliated to Cancer Hospital Chinese Academy of Medical Sciences/Cancer Hospital Affiliated to Shanxi Medical University Taiyuan Shanxi China

**Keywords:** cell metabolism, Epstein–Barr virus, glycolysis, immune evasion, lung cancer

## Abstract

Epstein–Barr virus infection has been implicated in various cancers, including lung cancer, where it influences cellular metabolism to promote tumorigenesis. This review examines the complex interplay between Epstein–Barr virus and cell metabolism in lung cancer, highlighting viral mechanisms of metabolic reprogramming and their implications for therapeutic strategies. Key viral proteins such as LMP1 and LMP2A manipulate glycolysis, glutaminolysis and lipid metabolism to support viral replication and immune evasion within the tumour microenvironment. Understanding these interactions provides insights into novel therapeutic approaches targeting viral‐induced metabolic vulnerabilities in Epstein–Barr virus‐associated lung cancer.

## 1 INTRODUCTION

Lung cancer is the leading cause of cancer‐related deaths globally, with approximately 1.8 million deaths annually.^1^ Despite advancements in detection and treatment, the prognosis for lung cancer patients remains poor, with a 5‐year survival rate of less than 17.8%.^2^ The heterogeneity of lung cancer, consisting primary of non‐small cell lung cancer (NSCLC) and small cell lung cancer (SCLC), presents significant challenges in understanding and managing the disease.^3^ Epstein–Barr virus (EBV), a ubiquitous gamma‐herpesvirus, infects over 90% of the global population, usually establishing a lifelong latent infection in B lymphocytes. While EBV is often asymptomatic in healthy individuals, it is well‐recognized for its role in the pathogenesis of several malignancies, including nasopharyngeal carcinoma, Burkitt's lymphoma and certain types of gastric carcinoma. Emerging evidence indicates that EBV may play a role in the aetiology of lung cancer, particularly in the context of immune suppression or genetic predisposition.^4^

The oncogenic potential of EBV is attributed to its ability to manipulate host cellular processes through the expression of viral proteins such as latent membrane protein 1 (LMP1), latent membrane protein 2 (LMP2) and Epstein–Barr nuclear antigen 1 (EBNA1). These proteins interfere with cell signalling pathways, promoting cellular proliferation, inhibiting apoptosis and modulating immune responses.^5^ The exact mechanisms by which EBV influences lung cancer development, however, remain incompletely understood. A critical aspect of cancer biology is the reprogramming of cellular metabolism to support rapid cell growth and proliferation. Cancer cells exhibit altered metabolic pathways, commonly referred to as the Warburg effect, a metabolic shift where cancer cells predominantly produce energy through glycolysis even in the presence of oxygen, is a hallmark of cancer metabolism. This metabolic reprogramming provides cancer cells with the necessary energy and biosynthetic precursors to sustain their growth.^5^ The interplay between oncogenic viruses and host cell metabolism has garnered increasing attention, as viruses like EBV can hijack host metabolic pathways to facilitate their replication and promote oncogenesis.^6^ This review aims to elucidate the intricate relationship between EBV infection and metabolic reprogramming in lung cancer. By examining the molecular mechanisms by which EBV alters cellular metabolism and contributes to lung cancer pathogenesis, we hope to provide insights into potential therapeutic strategies that target these pathways.

## 2 EPSTEIN–BARR VIRUS

### 2.1 Biology of EBV

EBV is a member of the *Herpesviridae* family, specifically classified as a gamma‐herpesvirus. It has a double‐stranded DNA genome of approximately 172 kilobases, encapsulated within an icosahedral protein capsid. This capsid is surrounded by a tegument layer and a lipid envelope, which contains viral glycoproteins essential for the infection of host cells.^7^

EBV primarily targets B lymphocytes, epithelial cells and occasionally T cells and natural killer (NK) cells. The infection begins when the viral envelope glycoprotein gp350 binds to the complement receptor type 2 (CR2 or CD21) on the surface of B cells, facilitating viral entry. In epithelial cells, entry is mediated by the interaction of viral glycoprotein B (gB) and gH/gL with cellular integrins.^8^ Once inside the host cell, EBV can establish two distinct types of infection: lytic and latent. In the lytic cycle, the virus undergoes active replication, producing new viral particles that are released to infect additional cells. This phase is characterized by the expression of immediate‐early, early and late viral genes, culminating in cell lysis. In contrast, during latent infection, the viral genome persists as an episome within the host cell nucleus, and only a limited set of viral genes is expressed.^9^ These latent genes, including EBNA1, LMP1, LMP2 and the EBV‐encoded small RNAs (EBERs), contribute to the maintenance of the viral genome and the transformation of host cells.^10^

### 2.2 EBV and oncogenesis

EBV is a potent oncogenic virus, with its transformative capabilities linked to its ability to manipulate host cellular processes. The virus is associated with several malignancies, including nasopharyngeal carcinoma, Burkitt's lymphoma, Hodgkin's lymphoma and gastric carcinoma. Its role in these cancers is mediated by the expression of specific latent and lytic genes that interfere with normal cellular functions.^11^ LMP1 acts as a constitutively active receptor that mimics CD40 signalling, activating the NF‐κB, JAK/STAT and MAPK pathways. This leads to increased cell proliferation, resistance to apoptosis and enhanced migration and invasion capabilities. LMP1 also induces the expression of various cytokines and chemokines, contributing to a pro‐inflammatory microenvironment that supports tumour growth.^12^ LMP2 has two isoforms, LMP2A and LMP2B, both of which play roles in maintaining EBV latency and promoting cell survival. LMP2A mimics B‐cell receptor signalling, providing survival signals in the absence of external stimuli, thereby preventing apoptosis.^13^ It also activates the PI3K/Akt pathway, which is crucial for cell survival and metabolic regulation.^12^ EBNA1 is essential for the replication and maintenance of the viral episome in latently infected cells. It also plays a role in cell transformation by interfering with the p53 and Rb tumour suppressor pathways, facilitating genomic instability and uncontrolled cell proliferation.^14^

EBERs are highly expressed in latently infected cells and have been implicated in modulating the host immune response. They inhibit interferon responses and activate the RIG‐I signalling pathway, contributing to the immune evasion and survival of infected cells. The transformative effects of EBV are not limited to the expression of individual viral proteins but involve the intricate interplay of multiple signalling pathways. The virus can modulate key cellular processes such as apoptosis, cell cycle regulation and immune surveillance. EBV‐induced epigenetic modifications also play a role in the reprogramming of host gene expression, further promoting oncogenesis.^15^

## 3 EBV AND LUNG CANCER

### 3.1 Epidemiology and clinical evidence

While EBV is widely recognized for its association with lymphoid and epithelial malignancies, its role in lung cancer has garnered increasing interest. The epidemiological evidence linking EBV to lung cancer is not as robust as for other EBV‐associated cancers, yet several studies have identified EBV DNA and proteins in lung cancer tissues, suggesting a potential role in lung carcinogenesis.^16^ Investigations into the prevalence of EBV in lung cancer patients have yielded varying results, likely due to differences in geographical regions, detection methods and patient populations. In certain studies, particularly those conducted in Asian populations, EBV DNA has been detected in a 4.7% proportion of lung cancer tissues.^17^

EBV‐associated lung cancers do not present with unique clinical features distinct from other forms of lung cancer. However, the presence of EBV can be determined through molecular diagnostic techniques, such as polymerase chain reaction (PCR) for EBV DNA, in situ hybridization for EBER, and immunohistochemistry for EBV‐specific proteins. These methods are crucial for identifying EBV involvement in lung cancer and differentiating it from other etiologies.^18^

### 3.2 Molecular mechanisms

The molecular mechanisms by which EBV contributes to lung cancer development are complex and multifaceted, involving both direct effects of viral proteins and indirect effects through modulation of the host cellular environment. EBV's oncogenic potential in lung epithelial cells is mediated by several key viral proteins. LMP1, LMP2 and EBNA1, which are typically expressed in latently infected cells, play pivotal roles in transforming host cells and promoting oncogenesis.^19^

In lung epithelial cells, LMP1 functions as an oncogenic driver by activating multiple signalling pathways, including NF‐κB, PI3K/Akt and MAPK.^20^ These pathways promote cellular proliferation, inhibit apoptosis and enhance the invasive properties of lung cancer cells. LMP1 also induces the expression of angiogenic factors and matrix metalloproteinases (MMPs), contributing to tumour progression and metastasis.^21^ LMP2A mimics B‐cell receptor signalling and activates the PI3K/Akt pathway in lung epithelial cells, providing survival signals that prevent apoptosis. This anti‐apoptotic effect is particularly important in the early stages of lung cancer development, allowing infected cells to evade cell death mechanisms and accumulate genetic mutations.^20^

EBNA1 plays a crucial role in maintaining the viral episome and regulating the expression of other latent genes. In lung cancer cells, EBNA1 has been shown to interact with the p53 tumour suppressor pathway, leading to reduced p53 activity and increased cell survival.^22^ Additionally, EBNA1 can induce genomic instability by promoting oxidative stress and DNA damage.^23^ EBV infection in lung epithelial cells can create a pro‐inflammatory microenvironment conducive to cancer development.^13^ LMP1‐induced expression of cytokines and chemokines recruits immune cells to the tumour site, which can contribute to chronic inflammation and promote tumour growth.^24^ Moreover, EBV can evade immune surveillance by downregulating major histocompatibility complex (MHC) molecules and inhibiting the activity of cytotoxic T cells, allowing infected cells to persist and proliferate.^25^ EBV can induce epigenetic changes in host cells, such as DNA methylation and histone modifications, which can lead to the silencing of tumour suppressor genes and activation of oncogenes. These epigenetic alterations are critical for the initiation and progression of lung cancer and represent a key mechanism by which EBV contributes to carcinogenesis.^26^ The interplay between EBV and other risk factors, such as smoking, environmental pollutants and genetic predispositions, can further exacerbate lung cancer development. EBV infection may act synergistically with these factors to enhance mutagenesis, disrupt cellular homeostasis and accelerate tumour progression.^27^

## 4 CELLULAR METABOLISM IN CANCER

### 4.1 Overview of cancer metabolism

Cancer cells undergo profound metabolic reprogramming to meet the demands of rapid proliferation, survival in hostile environments and metastasis. This metabolic shift is a hallmark of cancer, involving alterations in various metabolic pathways that distinguish cancer cells from their normal counterparts. One of the most well‐known metabolic changes in cancer cells is the Warburg effect, described by Otto Warburg in the 1920s.^28^ This phenomenon is characterized by an increased reliance on glycolysis for energy production, even in the presence of sufficient oxygen for oxidative phosphorylation (OXPHOS).^29^ Cancer cells convert glucose to lactate through aerobic glycolysis, allowing them to rapidly generate ATP and metabolic intermediates necessary for biosynthetic processes.^30^ This shift provides cancer cells with a growth advantage by enabling the efficient production of macromolecules required for cell proliferation. While glycolysis is a central feature of cancer metabolism, cancer cells also exhibit alterations in other metabolic pathways.^31^ The pentose phosphate pathway (PPP) is often upregulated to provide ribose‐5‐phosphate for nucleotide synthesis and NADPH for reductive biosynthesis and antioxidant defence.^32^ Additionally, the tricarboxylic acid (TCA) cycle and glutaminolysis are reprogrammed to support the anabolic needs of cancer cells. Glutamine, in particular, becomes a critical nutrient, serving as a carbon and nitrogen source for the synthesis of nucleotides, amino acids and lipids.^33^

### 4.2 Metabolic reprogramming in cancer

Cancer cells frequently exhibit changes in the expression and activity of key metabolic enzymes. For example, hexokinase 2 (HK2), which catalyses the first step of glycolysis, is often overexpressed in cancer cells, enhancing glucose uptake and metabolism. Similarly, pyruvate kinase M2 (PKM2), which regulates the final step of glycolysis, is upregulated and can exist in a dimeric form that favours the accumulation of glycolytic intermediates for biosynthesis.^34^ Another critical enzyme, isocitrate dehydrogenase (IDH), is frequently mutated in certain cancers, leading to the production of the oncometabolite 2‐hydroxyglutarate (2‐HG).^35^ 2‐HG inhibits α‐ketoglutarate‐dependent dioxygenases, resulting in altered epigenetic regulation and promoting tumorigenesis.^36^ The metabolic reprogramming of cancer cells is driven by oncogenes and tumour suppressors. For instance, the oncogene c‐Myc upregulates the expression of glycolytic enzymes, glutamine transporters and enzymes involved in nucleotide biosynthesis.^37^ Hypoxia‐inducible factor 1‐alpha (HIF‐1α), stabilized under hypoxic conditions common in tumours, promotes glycolysis and suppresses mitochondrial respiration.^38^ Conversely, the tumour suppressor p53 plays a key role in maintaining metabolic homeostasis. p53 regulates genes involved in OXPHOS, glycolysis and antioxidant defence.^39^ Loss of p53 function, common in many cancers, leads to increased glycolysis, reduced mitochondrial function and enhanced oxidative stress.^40^

Oncometabolites are metabolic intermediates that accumulate in cancer cells and contribute to oncogenesis. Besides 2‐HG produced by mutant IDH, other oncometabolites include succinate and fumarate, which accumulate due to mutations in succinate dehydrogenase (SDH) and fumarate hydratase (FH), respectively.^36^, ^41^ These oncometabolites inhibit α‐ketoglutarate‐dependent dioxygenases, leading to epigenetic alterations and stabilization of HIF‐1α, promoting a pseudo‐hypoxic state that drives cancer progression.^41^ Although cancer cells often rely on glycolysis, mitochondria remain crucial for cancer metabolism.^42^ The TCA cycle provides precursors for biosynthesis, and mitochondria are involved in regulating apoptosis.^43^ Cancer cells can exhibit altered mitochondrial dynamics, such as changes in mitochondrial fusion and fission, to adapt to metabolic stress and support tumour growth.^44^

The tumour microenvironment (TME) exerts significant influence on cancer cell metabolism.^45^ Hypoxia, nutrient deprivation and acidic conditions within the TME drive metabolic adaptations that enhance cancer cell survival and proliferation.^46^ Hypoxia, for example, induces HIF‐1α, which shifts cellular metabolism towards glycolysis and angiogenesis.^47^ Additionally, interactions with stromal cells, immune cells and extracellular matrix components can modulate metabolic pathways in cancer cells.^48^, ^49^

## 5 THE INTERPLAY BETWEEN EBV AND CELL METABOLISM IN LUNG CANCER

### 5.1 Metabolic reprogramming by EBV

EBV significantly influences the metabolic landscape of infected cells, driving reprogramming that supports oncogenesis. In the context of lung cancer, EBV's interaction with cellular metabolism creates a microenvironment conducive to tumour development and progression.^50^ EBV infection in lung epithelial cells leads to the upregulation of glycolysis, reminiscent of the Warburg effect observed in many cancers. The viral protein LMP1 is a key player in this process, enhancing glucose uptake and glycolytic flux by upregulating glucose transporters (e.g. GLUT1) and glycolytic enzymes (e.g. HK2 and PKM2).^51^ Concurrently, LMP1‐induced activation of the NF‐κB and PI3K/Akt pathways further promotes glycolysis while also supporting mitochondrial biogenesis and function, ensuring a balance between glycolytic and oxidative metabolism.^52^, ^53^, ^54^

EBV modulates key metabolic regulators such as c‐Myc and HIF‐1α. LMP1 and LMP2A activate c‐Myc, which in turn enhances the expression of glycolytic enzymes and glutaminolysis pathways.^54^, ^55^ HIF‐1α stabilization under hypoxic conditions, often exacerbated by EBV‐induced inflammation, shifts cellular metabolism towards glycolysis and away from oxidative phosphorylation, aiding in the survival and proliferation of EBV‐infected lung cancer cells.^56^, ^57^ EBV infection also reprograms glutamine metabolism, making it a crucial nutrient for cancer cell survival. LMP1 and LMP2A promote the expression of glutaminase, increasing the conversion of glutamine to glutamate, which feeds into the TCA cycle and supports anabolic processes^54^ (Table.1). Additionally, EBV influences lipid metabolism by upregulating fatty acid synthesis pathways, providing essential components for membrane biogenesis and energy storage.

**TABLE 1:** EBV influence on metabolic pathways in lung cancer.

Metabolic pathway	EBV effect	Viral proteins	Mechanisms and pathways activated
Glycolysis	Upregulation; Increased expression of glucose transporters (e.g. GLUT1) and glycolytic enzymes (e.g., HK2 and PKM2)	LMP1, LMP2A	Activation of NF‐κB, PI3K/Akt pathways
Glutaminolysis	Enhanced; Increased expression of glutaminase	LMP1, LMP2A	Promotion of glutamine to glutamate conversion
Lipid metabolism	Upregulated; Increased fatty acid synthesis pathways	LMP1	Enhanced lipid membrane biogenesis and energy storage
Mitochondrial biogenesis	Supported; Activation of mitochondrial biogenesis	LMP1	Maintenance of metabolic balance between glycolysis and oxidative phosphorylation

### 5.2 Immune evasion and metabolism

EBV's ability to evade the host immune system is intricately linked to its modulation of cellular metabolism. The virus‐induced metabolic reprogramming creates a microenvironment that impairs immune cell function.^58^ For instance, the lactate produced during increased glycolysis can suppress the activity of cytotoxic T cells and natural killer cells, which are crucial for the immune response against cancer.^59^ The metabolic state of the host cell influences EBV latency and reactivation.^9^ Nutrient availability and metabolic signals can trigger the switch from latent to lytic infection.^60^ This dynamic interplay ensures that EBV can exploit favourable metabolic conditions to enhance its replication and spread, further contributing to tumour progression.^61^

## 6 THERAPEUTIC IMPLICATIONS

### 6.1 Targeting EBV in lung cancer

Direct targeting of EBV with antiviral agents, such as ganciclovir and acyclovir, can inhibit viral replication and reduce the viral load in infected cells.^62^ However, the efficacy of these treatments in EBV‐associated lung cancer is limited, as the virus predominantly exists in a latent state in these cells.^63^ More advanced strategies, such as lytic induction therapy combined with antiviral drugs, aim to reactivate latent EBV and subsequently eliminate infected cells through antiviral agents or immune‐mediated mechanisms.^64^ Immunotherapeutic approaches, including adoptive T cell therapy and immune checkpoint inhibitors, show promise in targeting EBV‐associated malignancies.^63^ Adoptive T cell therapy involves the infusion of autologous or allogeneic T cells specific for EBV antigens, aiming to boost the immune response against EBV‐infected cancer cells.^65^ Immune checkpoint inhibitors, such as PD‐1/PD‐L1 and CTLA‐4 blockers, can enhance the anti‐tumour immune response by relieving the suppression imposed by EBV‐induced immune evasion mechanisms.^66^

### 6.2 Metabolic targets in EBV‐positive lung cancer

Given the reliance of EBV‐infected lung cancer cells on glycolysis, targeting key glycolytic enzymes offers a therapeutic strategy. Inhibitors of HK2, such as 2‐deoxyglucose (2‐DG) and PKM2 inhibitors can disrupt the glycolytic flux, impairing energy production and biosynthesis in cancer cells.^67^ Additionally, blocking lactate dehydrogenase (LDH) can reduce lactate production, mitigating its immunosuppressive effects.^68^ Targeting glutamine metabolism presents another viable approach.^69^ Inhibitors of glutaminase, such as CB‐839, can deplete the glutamine pool, disrupting the TCA cycle and anabolic processes in EBV‐positive lung cancer cells. This strategy can induce metabolic stress and reduce tumour growth.^70^, ^71^, ^72^

Since EBV also influences lipid metabolism, targeting fatty acid synthesis enzymes, such as acetyl‐CoA carboxylase (ACC) and fatty acid synthase (FASN), can impair the production of essential lipids required for cell membrane integrity and signalling. This approach can reduce the proliferation and survival of EBV‐infected lung cancer cells.^73^ Combining metabolic inhibitors with existing therapies holds significant promise. For instance, combining glycolysis or glutaminase inhibitors with immune checkpoint inhibitors could enhance the anti‐tumour immune response while disrupting the metabolic support of cancer cells.^74^, ^75^, ^76^, ^77^ Additionally, combining antiviral therapies with metabolic modulators might effectively target both the viral and metabolic aspects of EBV‐positive lung cancer.^71^, ^76^

## 7 CONCLUSION

The interplay between EBV and cellular metabolism represents a promising avenue for understanding the pathogenesis of EBV‐associated lung cancer. Viral proteins such as LMP1 and LMP2A orchestrate metabolic reprogramming in infected cells, fostering a pro‐tumorigenic environment characterized by enhanced glycolysis, glutaminolysis and altered lipid metabolism. Therapeutic strategies targeting these metabolic vulnerabilities, alongside antiviral and immunotherapeutic approaches, hold the potential for improving outcomes in EBV‐positive lung cancer patients. Future research should focus on elucidating specific mechanisms of EBV‐induced metabolic dysregulation and translating these findings into effective clinical interventions.

## AUTHOR CONTRIBUTIONS

**Hongwei Wang:** Conceptualization (lead); supervision (lead).

## CONFLICT OF INTEREST STATEMENT

The authors declare no conflict of interest.

## Data Availability

Data sharing not applicable to this article as no datasets were generated or analysed during the current study.
